# Regulation via compartmentation: adaptive localization of the proteasome under stress

**DOI:** 10.1042/BST20253082

**Published:** 2025-12-11

**Authors:** Aaron Ciechanover, Ido Livneh

**Affiliations:** 1 The Rappaport Technion Integrated Cancer Center (R-TICC) and the Rappaport Faculty of Medicine and Research Institute, Technion-Israel Institute of Technology, Haifa, 3109601, Israel; 2 Institute of Pathology and Cytology, Rambam Health Care Campus, Haifa, 3109601, Israel

**Keywords:** ubiquitin proteasome system, proteostasis, stress response, proteasomes, ubiquitin signaling, proteasome dynamics, proteasomes, proteostasis, stress response, ubiquitin proteasome system, ubiquitin signaling

## Abstract

Protein degradation by the ubiquitin-proteasome system and autophagy are essential mechanisms that are involved in virtually all cellular activities, and their inadequate function was shown to underlie the pathogenesis of various medical conditions. Much of the study into these proteolytic systems has been focused on the components that facilitate the selective substrate identification and targeting for degradation. Given that most of the specific breakdown of proteins is mediated via their modification by ubiquitin, much research was dedicated to the enzymes which are responsible for substrate recognition and ubiquitination—E3 ubiquitin ligases. In addition to the complexity of substrate recognition and targeting for degradation, the mechanisms governing proteasome function were found to be tightly regulated, including the assembly of the different proteasomal sub-complexes, its different compositions and specialized subtypes such as the immunoproteasome, posttranslational modification of proteasomal subunits, and adaptations in its activity in face of different cellular states and stress conditions. Studies from recent years have highlighted an as-yet unexplored tier of proteasome regulation, namely its subcellular compartmentation and trafficking. Intracellular proteasome shuttling was shown to serve as an essential stress-coping mechanism in tumor cells and is emerging as a potential target for therapeutic interventions.

## Introduction

Protein turnover is a tightly regulated process, the importance of which is underscored by the diseases resulting from its dysregulation. Inadequate protein removal and subsequent aggregation underlie the pathogenesis of neurodegenerative diseases, while destabilization of tumor suppressors is involved in the pathogenesis of different malignancies [1,2]. The ubiquitin–proteasome system (UPS) is responsible for the majority of selective protein degradation, alongside autophagy, which can selectively remove organelles through macroautophagy and certain soluble proteins via chaperone- and receptor-mediated autophagy. In addition, autophagy removes cytoplasmic proteins in a nonspecific manner. Selective targeting of proteins for degradation by either the UPS or autophagy is facilitated, to a large extent, via their ubiquitination, i.e., their posttranslational modification with one or more ubiquitin moieties [3]. In accordance, the selective and timely removal of protein substrates by the UPS stems from their recognition by specific ubiquitin ligases, which comprise the largest group of proteins within the system with hundreds of different members [4]. Newly identified E3 ubiquitin ligases continue to emerge, and the number of known ligases is already greater than that of kinases within the human genome [5]. Importantly, the use of the ubiquitin mark for protein removal is highly conserved and can be found in all eukaryotic cells, and similar systems for substrate tagging and degradation were identified also in certain bacteria [6,7].

**Figure 1:**
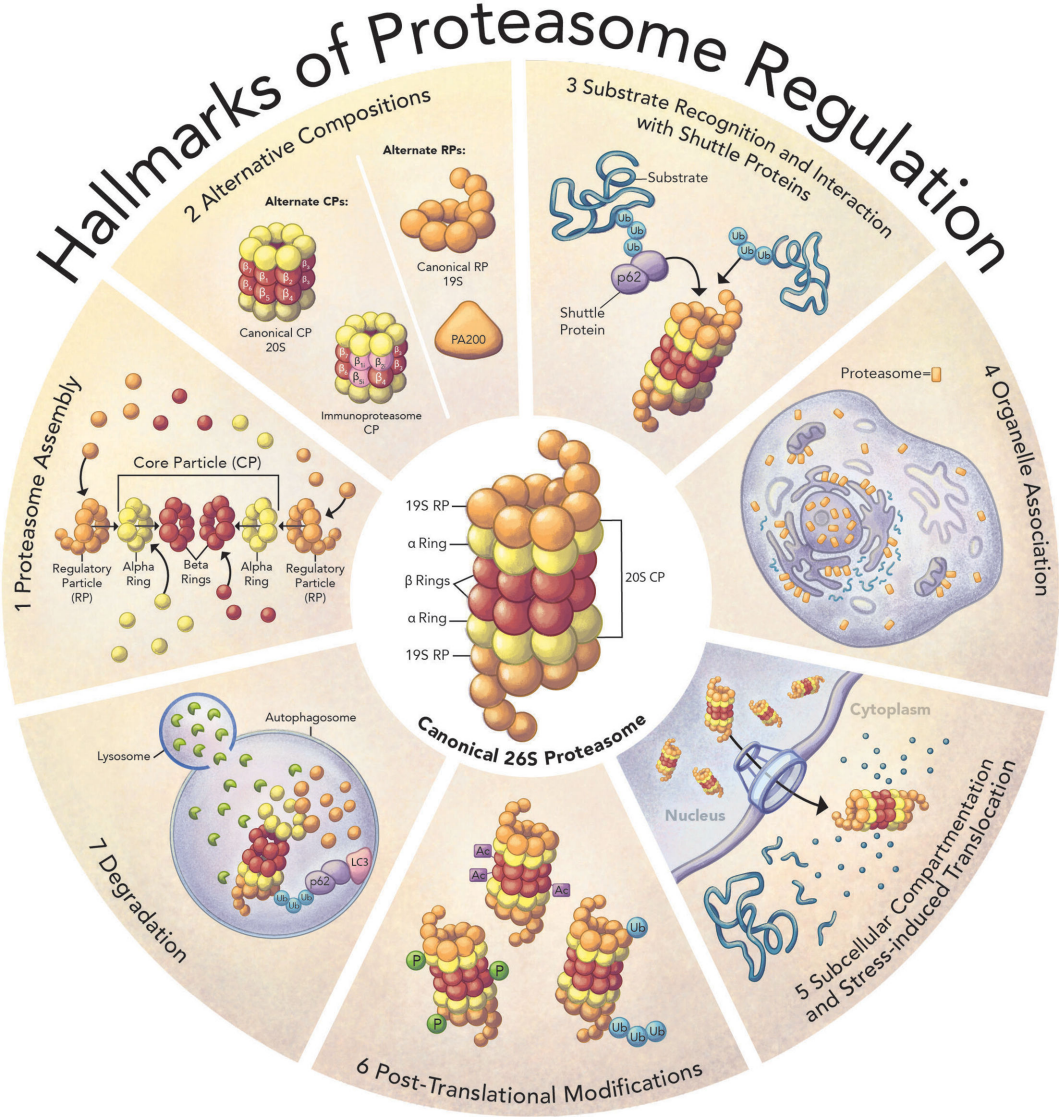
Hallmarks of Proteasome Regulation. Presented are major mechanisms through which the 26S proteasome is regulated, which consequently affects various cellular processes. See Main Text for further details. Schematically depicted are [1] the assembly of the 26S proteasome from its subunits [2]; Different types of proteasomes, including variations in either subunits of the catalytic particle (CP, 20S; e.g., immunoproteasome) and regulatory particles (19S, RP; e.g., the alternative PA200 RP) [3]; recognition of ubiquitinated substrates targeted to the proteasome either directly or via following interaction with shuttle proteins such as p62 [4]; proteasome activity linked with specific organelles and functions, such as ER-associated degradation (ERAD) of ER-retro-translocated unfolded proteins, and axonal transport of proteasomes to maintain synapse proteostasis [5]; dynamic distribution of the 26S proteasome between the different cellular compartments, mostly between the nucleus and the cytosol, which is a response to stress cues (e.g., amino acid starvation, hypoxia) [6]; different post-translational modifications of the proteasome, the function of which is still largely elusive for most of these modifications; and last[7], the predator becomes the prey: degradation of the 26S proteasome by autophagy.

While this group of substrate-recognizing E3 ligases is rather specific, both earlier and later events in the cascade which subsequently lead to protein breakdown are common to all substrates. Prior to its conjugation to proteins, ubiquitin must be activated by E1—a ubiquitin-activating enzyme. Unlike the numerous E3 ubiquitin ligases, there is only one E1 ubiquitin activating enzyme (or arguably two [8]), which generates active ubiquitin moieties for the modification of all of its substrates [9]. Similarly, the proteolytic breakdown of ubiquitinated proteins into peptides is carried out by the proteasome, a ~ 2.5 MDa complex composed of more than 30 subunits [10]. While the proteasome selectively recognizes ubiquitinated protein substrates, it is presumably ‘agnostic’ to the identity of the proteins themselves, as they were already identified specifically in the ubiquitination process. This two-step mechanism was essential for the evolutionary evolvement of a highly selective and specific system that will recognize and remove a single protein molecule (out of the entire population of otherwise identical protein) that was denatured or mutated, for example, or an entire cohort of the same protein (e.g., cyclin A) while preserving the entire cellular proteome. This is the only known case of a cryptic protease that recognizes a post-translational modification. The uncontrolled activity of all other known proteases is restricted by a physical/biochemical barrier (membrane and low pH for lysosomal enzymes, mucinous secretion by the gastrointestinal tract, and secretion of tissue factors that limit blood coagulation to the injury site). Nevertheless, studies from recent decades have unraveled multiple layers of proteasomal regulation, which are essential for adequate protein quality control (PQC) and virtually all cellular functions [11] (Figure 1).

### Regulatory mechanisms of proteasome function

Much of the research into the proteasome was dedicated to mechanistic understanding of its composition and assembly [12]. As mentioned, the 26S proteasome is a complex composed of a large number ( > 30) of subunits, which in turn form subcomplexes responsible for the different steps of the removal of a ubiquitinated protein substrate. The central subcomplex of the proteasome is the 20S catalytic (or core) particle (CP), which includes all of the complex’s proteolytic sites, accountable for substrate breakdown into short peptides. Yet, this final step is made possible only following substrate recognition through proteasomal ubiquitin receptors which bind ubiquitin moieties that are bound to the protein substrate, unfolding of the latter and its insertion into the barrel-shaped CP. These preparative steps are carried out by the subunits of the 19S proteasome subcomplex, or regulatory particle (RP) [13,14]. The RP is in fact larger and is composed of a greater number of subunits compared with the CP, highlighting the importance of the different steps which precede the catalytic breakdown of peptide bonds [15]. Further, in the ‘canonical’ proteasome (i.e., the fully assembled holoenzyme), each 20 S CP is ‘capped’ by two RPs, one on each of its termini, allowing, in principle, entry of substrates to the catalytic chamber from either side. How this mega-Da complex is assembled is the topic of many studies, which have unraveled both the order in which the subunits are being organized into the different subcomplexes, as well as ancillary chaperones which are essential for the formation of the functional holoenzyme [16–20].

Interestingly, while much of the above studies focused on the ‘canonical’ proteasome composed of one 20 S CP and two 19S RPs flanking the former from both of its sides, there are ‘alternative’ species of proteasome that were found to co-exist in the same cells as the ‘canonical’ one. First, while the 19S proteasome subcomplex is the most studied RP, there are in fact other RPs that can cap the 20 S CP. PA28 and PA200 are additional complexes that can bind the 20S proteasome, as does PA200, an RP composed of a single protein [21,22]. Interestingly, many 20 S CP molecules can be found uncapped at a given time [23], which together with the long half-life of the proteasome [24], and the fact that an uncapped 20 S CP is largely inactive, suggest that there is a reservoir of the pre-assembled CPs that can be capped by RPs in response to changing cellular requirements, facilitating fine-tuning of PQC [25]. Some subunits of the 20 S CP were also shown to be interchangeable. The catalytic subunits 1, 2, and 5 can be replaced by alternative subunits, named 1i, 2i, and 5i, respectively, where the ‘i' stands for *immune* [26]. These immune subunits, encoded by different genes than the ‘canonical’ or *constitutive* proteasome subunits, are also catalytic, yet their breakdown of proteins yields peptides that are better suited for binding to and presentation by the major histocompatibility complex (MHC) class I [27,28]. Peptidases involved in processing proteasome-derived peptides into amino acids were suggested to contribute also to antigen presentation [29]. Interestingly, antigenic peptides were shown to distribute between the nucleus and cytoplasm in a manner affecting their presentation by MHC class I [30]. As expected, the level of immunoproteasome (iProteasome) is up-regulated by proinflammatory cytokines such as interferon, but other stress conditions, such as oxidative stress, were also shown to stimulate the formation of the iProteasome, underscoring its role not only against foreign intruders via presentation of their antigens but also under other cellular stress conditions [31,32].

Besides the multiple compositions made possible by the different 20S CPs and RPs, the proteasome was also shown to be heavily modified by posttranslational modifications [33]. Intriguingly, while numerous PTMs of the proteasome were identified, little is known on the role(s) and the factors that regulate most of them. The ubiquitination of certain subunits was suggested to facilitate the degradation of the complex by autophagy, while the exact function of some ubiquitination sites within the proteasome has remained elusive [34,35]. The same is true for the numerous phosphorylation sites which were identified on various proteasomal subunits, mostly via proteomic studies [36]. Even specific insights that were gained or verified concerning the phosphorylation of certain subunits are, in many cases, fragmentary: it is either unclear which site within a subunit is phosphorylated, by which kinase, following what stimuli, or to what end [37,38]. All in all, while PTMs of the proteasome are abundant and are expected to modify its activity, our current functional and mechanistic understanding of these PTMs, as well as how they affect proteasome function and its cellular roles, is still limited. Newly identified roles of the proteasome, its involvement in cellular response to selective cues, and previously unknown interactions with other proteins (see below) may also shed light on the roles of some of the proteasomal PTMs.

For several decades, researchers have been studying the mechanisms regulating the proteasome role in degradation of cellular proteins, while the mechanism(s) that underlies the removal of the proteasome—by itself composed of proteins—has remained open. Degradation of the proteasome is co-ordinated with its assembly, given that free proteasomal subunits are hardly found in cells, which raises a question concerning the fate of unassembled subunits. It was found that subunits that are synthesized in excess (i.e., relative to their required stoichiometric amounts) are degraded by the proteasome itself, which thereby regulates its own components [39]. With regard to the assembled proteasome, it was found to be removed via autophagic degradation, also termed proteaphagy, a mechanism conserved in plants, yeast, and mammalian cells [40–42].

### Specific proteasomal functions

While the proteasome is not the link in the UPS cascade chain where substrate specific recognition is achieved, a growing body of evidence has underscored that proteasome function is closely responsive to specific conditions and signals. Proteasomal adaptation to such stimuli may affect the identity of the proteins which are degraded under different physiological and pathological states [43], in concert with the E3 ubiquitin ligases which facilitate the substrate-specific ubiquitination. Endoplasmic reticulum-associated degradation (ERAD) is a prototypic example for a case in which an E3 ubiquitin ligase is associated with a certain function (i.e., degradation of ER-inserted proteins) and not specific substrates. Such proteins are ubiquitinated by dedicated ER-associated E3 ligases (e.g., VCP/p97 and HRD1) and are removed by adjacent proteasomes [44,45].

In other cases, role-specific proteasome activity is achieved via shuttle proteins, some of which serve as extra-proteasomal ubiquitin receptors that identify proteins destined for degradation and deliver them to the proteasome. For example, Ubiquilin-4 (UBQLN4) is a shuttle protein that targets substrates for proteasomal degradation, a role that facilitates an adequate cellular response to DNA damage [46]. Another shuttle protein, RAD23B, is required for the formation of nuclear condensates which contain the proteasome, among other proteins, in response to hyperosmotic stress [47].

Somewhat similarly, the shuttle protein SQSTM1/p62 is essential for the formation of cytoplasmic condensates of UPS components, including the proteasome, in which substrate removal is taking place [48]. These degradation foci are not bound by membranes, but are rather separated from their surroundings via liquid-liquid phase separation (LLPS). Such a transient gathering of high concentration of the components of the UPS required for degradation of a given substrate (the conjugating enzymes, the proteasome, deubiquitinating enzymes, shuttle proteins, and specific substrates) represents a highly efficient, responsive, and selective PQC mechanism [49]. As a result, these LLPS condensates are drawing much interest in the field of protein degradation, as well as in other research communities.

The importance of adequate proteasome regulation was demonstrated also in neuronal function through its proteolytic roles in both neurons and Schwann cells, responsible for peripheral nerve myelination [43,50]. Similarly, proteasome function was also implicated in hereditary neuropathies [51]. Specifically in the context of its spatial regulation, proteasome transport along axons was shown to play an essential role in synapse maintenance and neuronal survival, via timely and spatially regulated PQC. Proteasome movement within neurons is facilitated by dynein, to which the proteasome is bound through PI31 that serves as an adaptor protein [52,53].

As can be deduced from the above examples of task-specific proteolytic activity, a key aspect of regulating proteasome activity lies in the localization of the catalytic complex, as well as in the formation of a local concentration of different components of the UPS that are required for the execution of protein degradation, including the proteasome. In agreement with this notion, we have found that the proteasome, despite being one of the largest soluble complexes within cells, is constantly moving between the different compartments to accommodate to the ever-changing pathophysiological conditions. Stress-induced translocation of the proteasome between the nucleus and the cytoplasm is an active process, facilitated by the nuclear pore complex (NPC) and exportins/importins [54]. Under normal physiological conditions, the proteasome can also redistribute into the nucleus as part of cell division, during which nuclear lamina are disintegrated, allowing the accumulation within newly forming nuclei without being transferred through the NPC [55]. Notably, while the proteasome can be shuttled through the NPC despite its considerable size [56], such active nucleo-cytoplasmic shuttling is energy-dependent, which means that constantly maintaining a dynamic steady state of proteasome distribution within cells is a ‘costly’ task. Given the evolutionary principle of energetic efficacy, one may conclude that this constant movement of the proteasome, which requires cellular energy, is also an important mechanism for adequate cellular function and/or survival.

### Stress-induced recruitment of preexisting proteasomes

**Figure 2:**
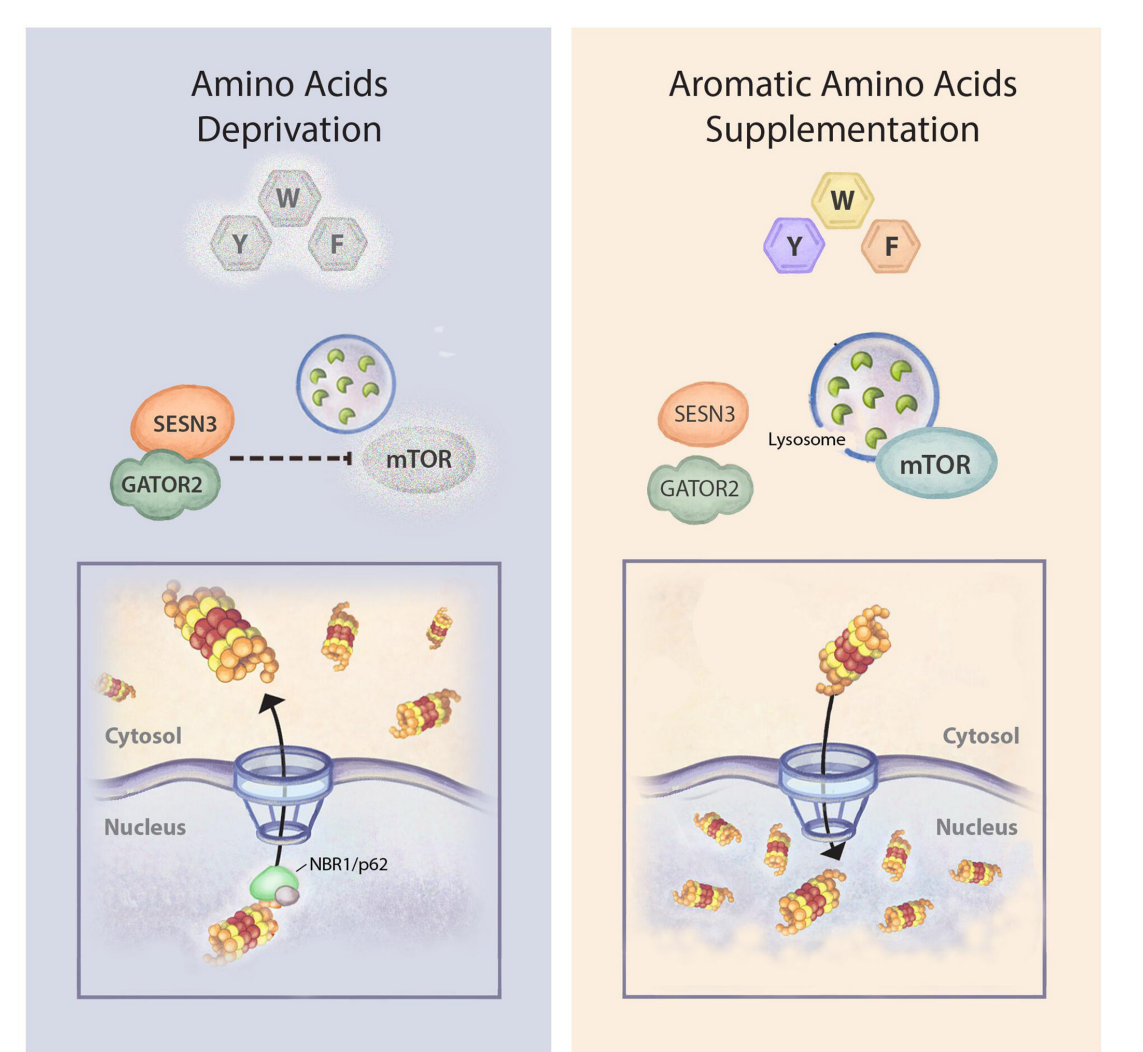
Aromatic amino acids regulate mTOR-dependent proteasome localization. During amino acid deprivation, inhibition of mTOR signaling promotes translocation of the proteasome from the nucleus to the cytosol via the shuttle proteins p62 and NBR1. Supplementation with the aromatic amino acids tyrosine, tryptophan, and phenylalanine (YWF) stimulates mTOR signaling, leading to sequestration of the proteasome within the nucleus. The YWF triad disrupts the inhibitory interaction between SESN3 and the GATOR2 complex, thereby restoring mTOR activity.

We have recently identified an as-yet undescribed regulatory mechanism of the UPS, where the proteasome is translocated from the nucleus to the cytosol in response to amino acid starvation. Under basal metabolic conditions, the proteasome is largely concentrated in the nucleus, whereas under shortage of amino acids, it is translocated to the cytosol. We identified mTOR as the underlying signaling pathway and showed that this stress-coping mechanism is governed by as yet undescribed mTOR-agonistic amino acids, the triad of the aromatic acids - Tyr, Trp, and Phe (Y, W, and F). In the presence of all amino acids (Control), the proteasome is mainly concentrated in the nucleus, whereas in the absence of all amino acids (Starvation), the proteasome is translocated from the nucleus to the cytoplasm. Supplementing the starved cells with the aromatic amino acids (Starvation + YWF) inhibited proteasome export, leading to its sequestration in the nucleus (Figure 2). In a complementary experiment, we showed that subtracting only YWF from the complete medium (‘-YWF’) is sufficient to stimulate proteasome translocation. Mechanistically, we found YWF to relay their signal upstream of mTOR via Sestrin3, disrupting its interaction with the GATOR2 complex. This, in turn, activates mTOR towards downstream substrates such as p62, a shuttle protein known to facilitate nucleo-cytoplasmic protein transfer [57]. Given the role of inadequate nuclear organization and trafficking in malignant states [58], we further studied the components involved in proteasome shuttling through the NPC, finding that p62, together with its homolog NBR1, is required for proteasome translocation (Figure 2), a movement that is also dependent on the NPC member protein NUP93, which was recently shown to enhance tumor aggressiveness [54,59].

To unravel the cellular function(s) of proteasome recruitment, we monitored protein breakdown following its translocation as well as its inhibition. We found that the proteasomal activity was stimulated ~ 2 fold, leading to destabilization of numerous proteins, 94% of which are cytosolic. This outcome was reversed by inhibiting proteasome translocation to the cytosol. Notably, it was shown that under mTOR inhibition (e.g., by Torin 1), ubiquitination is initially up-regulated, followed by a decrease in the level of the conjugates, mediated by their proteasomal removal [60]. We found that preventing proteasome export from the nucleus inhibited degradation and depletion of ubiquitin adducts, underscoring the central role of nuclear-originated proteasome in cytosolic ubiquitin-mediated proteolysis.

Importantly, while the cells can tolerate starvation to all 20 amino acids, a tolerance related largely to the export of the proteasome from the nucleus to the cytosol and the stimulation of degradation of cytosolic proteins, addition of YWF to the starving cells, thereby sequestering the proteasome to the nucleus, resulted in cell death [50]. Attempting to expand the biochemical context of proteasome dynamics, we showed that the recruitment of the proteasome from the nucleus occurs also in response to stress conditions other than amino acid starvation, such as hypoxia [61]. Given the variety of upstream governing signals, as well as the downstream affected phenotypes, we next looked for cellular functions that are affected by proteasome translocation and the YWF signal. Using RNA sequencing to study the effect of proteasome dynamics on various cellular pathways, we found that concomitantly with nuclear proteasome sequestration, the expression of transcripts associated with cell survival and viability was down-regulated, while those associated with functions such as apoptosis and necrosis were up-regulated. Evaluating additional cellular processes, we found that inhibiting proteasome recruitment down-regulated kinase- and phosphorylation-associated cascades, protein and carbohydrate metabolism, and other homeostatic pathways [61]. While a thorough understanding of the mechanisms linking proteasome dynamics with these pathways requires further research, our findings demonstrate that the roles of YWF-mediated proteasome dynamics are much broader than the context of amino acids metabolism only.

### Proteasome translocation to the cytosol promotes tumorigenesis

Planning to study proteasome dynamics in tumor models in animals, we realized that it is impossible to effectively starve tumors to amino acids *in vivo*, since a protein-poor diet will stimulate degradation of the organismal proteins and in particular proteins of skeletal muscles. Yet, our findings concerning the different stimuli that induce proteasome translocation pointed out that proteasome translocation is not dictated merely by the level of amino acids. Further, the identification of various cellular functions that are affected by proteasome translocation highlighted that the proteasome is recruited to the cytosol not only in order to supplement cells with amino acids from protein breakdown but is also serving additional functions. Therefore, proteasome recruitment probably embodies a stress-coping mechanism that cancer cells employ under a variety of pathophysiological conditions. For instance, a growing tumor is poorly perfused relative to normal tissues, generating hypoxia and shortage of nutrients. As a consequence of such multifactorial stress conditions experienced by solid tumors, and in light of the constant movement of the proteasome within cells in order to adapt to the different requirements, we hypothesized that the proteasome will localize mostly to the cytoplasm of tumor cells—without any exogenous perturbations. In addition, we hypothesized that if tumor cells are employing proteasome shuttling to the cytoplasm as an inherent stress-coping mechanism, it may turn out to be essential for their survival, as was found in the case of cultured cells [54].

Indeed, we found that in tumors, the proteasome is mostly cytosolic. Importantly, supplementing YWF to the surrounding of the tumor resulted in nuclear sequestration of the proteasome within tumor cells, and just like in cultured cells, led to apoptosis. The tumors treated with YWF to ‘lock’ the proteasome in their nuclei were significantly smaller and demonstrated areas of fibrosis, consistent with a treatment effect, such as the one observed in currently used drugs that are administered prior to surgical resections (e.g., in esophageal, colorectal, and gastric carcinomas) [62–64]. It should be noted that proteasome recruitment under stress, as well as its essentiality for survival, was demonstrated in cancer cells from different origins, as well as nonmalignant cells [54,61,65], pointing out that this phenomenon is characteristic of a broad array of tumors and tissues, thereby suggesting that nuclear sequestration of the proteasome is a potential *Achilles heel* that may be common to different types of cancer. In agreement with this notion, YWF-induced nuclear sequestration of the proteasome was found to be effective against different types of tumors, including both human-derived xenografts and endogenous mouse tumors [61].

**Perspectives**

- **Importance of the field:** The ubiquitin system is responsible for specific degradation of abnormal/misfolded/damaged/denatured proteins, the accumulation of which can result in diseases (e.g., cancer, NDD); as well as that of normal proteins that completed their function, and their presence is no longer needed - anand may be dangerous to cells (e.g., cell cycle regulators and transcription factors). Therefore, the regulation of the system and timely recognition of its substrates are crucial for the proper function of the cell.
- **Current thinking:** Proteasome regulation is multifaceted, involving dynamic assembly of the 26S complex, and the existence of specialized variants such as the immunoproteasome. Substrate delivery relies on both direct recognition and adaptor proteins like p62. Some key proteasomal functions are linked to organelle-specific roles (e.g., ERAD, synaptic maintenance), and the complex also shuttles between compartments in response to stress. Posttranslational modifications of proteasomal subunits add another layer, and the proteasome itself is subjected to degradation via autophagy.
- **Future directions:** What is currently known is mostly at the phenomenological level but is not tightly linked to the different functions of the proteasome under different pathophysiological conditions. For instance, while numerous posttranslational modifications of the proteasome were identified, the specific role of most of these modifications is still unclear. Also, much is still unknown concerning the requirement for ubiquitin chains, their length, structure, and mixture of bonds, and how they affect recognition by the proteasome.

## Open Access

This article has been published open access under our Subscribe to Open programme, made possible through the support of our subscribing institutions, learn more here: https://portlandpress.com/pages/open_access_options_and_prices#conditional

## Abbreviations

CP: core particle
ERAD: endoplasmic reticulum-associated degradation
LLPS: liquid-liquid phase separation
MHC: major histocompatibility complex
PQC: protein quality control
RP: regulatory particle
UPS: ubiquitin−proteasome system
